# Extended the depth of field and zoom microscope with varifocal lens

**DOI:** 10.1038/s41598-022-15166-x

**Published:** 2022-06-30

**Authors:** Yani Chen, Hang Liu, Yin Zhou, Feng-Lin Kuang, Lei Li

**Affiliations:** School of Electronics and Information Engineering, Sichuan University, Chengdu, 610065 China

**Keywords:** Optical imaging, Microscopy, Applied optics, Adaptive optics

## Abstract

Extending the depth of field (DOF) is especially essential in thick and 3D sample imaging. However, it's difficult to achieve both large DOF and high resolution in a zoom microscope. Currently, the use of optical sectioning to expand DOF still has the problem of inconstant magnification. Here, we develop an extended the depth of field (EDOF) and zoom microscope, which can realize EDOF with constant magnification and high resolution. Besides, the proposed microscope can achieve optical axial scanning at different NA and magnifications in real time without any mechanical movement. The proposed varifocal lens is employed to realize optical axial scanning, zooming, and keeping constant magnification when extending the DOF. Experimental results show that the proposed microscope can realize a continuous optical zoom of 10–40×, NA from 0.14 to 0.54, and the DOF of microscope can be extended to 1.2 mm.

## Introduction

In order to accurately obtain all the information of the observed object, extending the depth of field (DOF) and zooming have always been the research hotspots in the field of microscopy. The DOF of the microscope is determined by the correlation between the NA and magnification^1^. Generally speaking, obtaining the transverse and axial resolution resolution is accompanied by a loss of horizontal resolution. Therefore, it's difficult to achieve both large DOF and high resolution at various magnifications in a zoom microscope.

Numerous methods were proposed to extend the DOF of the microscope, including aperture coding^2,3^, pupil filter^4^ and wavefront coding^5,6^, etc. And in the practical applications, the most common method is the optical sectioning techniques, which collects the information of the continuous focal plane by scanning or zooming to reconstruct the object. The current microscopic techniques, such as wide-field microscopy^7^, confocal microscopy^8^, two-photon microscopy^9^ and light sheet microscopy^10^, can realize 3D imaging by this way. There are many ways to obtain optical slices. Traditional axial scanning method realizes axial focus scanning by changing the distance of the lens group in the microscope or moving the position of the stage^11,12^. Although easy to operate, mechanical disturbance may affect the stability of the microscope system and even cause the loss of observation objects. In order to break through the limitations of mechanical scanning, new zoom elements without mechanical movement are used to solve this problem. The multi-focal microlens array (MLA) can get information of different focal planes by switching the optical power^13,14^, which shows high spatial resolution and dynamic speed. However, crosstalk between different zones within lenslet and adjacent lenslets may affect the image quality. Besides, the aperture of the microlens array is relatively small, which limits its application in the microscope with high NA. The varifocal lens is a good alternative for not only axial scanning but optical zooming. The varifocal lens, including the electrowetting lens^15–20^, liquid crystal lens^21–23^, tunable acoustic gradient (TAG)^24^ and polymer lens^25,26^, can vary its focal length by changing the curvature or index of lens. The varifocal lenses have good performance in zoom speed and continuity, and can eliminate some negative effects of mechanical scanning. However, the problem of inconstant magnification still exists in in axial scanning. The issue of inconstant magnification directly affects the accuracy of image reconstruction. At present, there are some methods to correct this problem. For example, setting an electrically adjustable lens to the aperture stop of the microscope objective^27^ or using an adaptive lens with multiple adjustable surfaces^28^ can achieve it. Though inconstant magnification can be corrected, the small aperture and low optical power of the electrowetting lens is not suitable for microscopes with a high NA and large zoom range. In addition, it is difficult to use external components in the zoom microscope with limited working distance. At present, there is no integrated microscope that can achieve extension of DOF at different magnifications and correct the problem of inconstant magnification.

In this paper, we develop an EDOF and zoom microscope, which can realize EDOF with constant magnification and high resolution. Besides, the proposed microscope can realize optical axial scanning at different NA and magnifications in real time without any mechanical movement. As the core part, the EDOF objective includes four varifocal lenses, which are specially designed and fabricated to realize zoom, optical axial scanning, and keeping constant magnification. At present, there is no microscope with the same function and our microscope objective has many possibilities to be adapted to other microscopy.

The proposed microscope can achieve a continuous optical zoom of 10–40× and NA from 0.14 to 0.54. In addition, the DOF of microscope can be extended to 1.2 mm.

## Structure of the EDOF and zoom microscope

**Figure 1:**
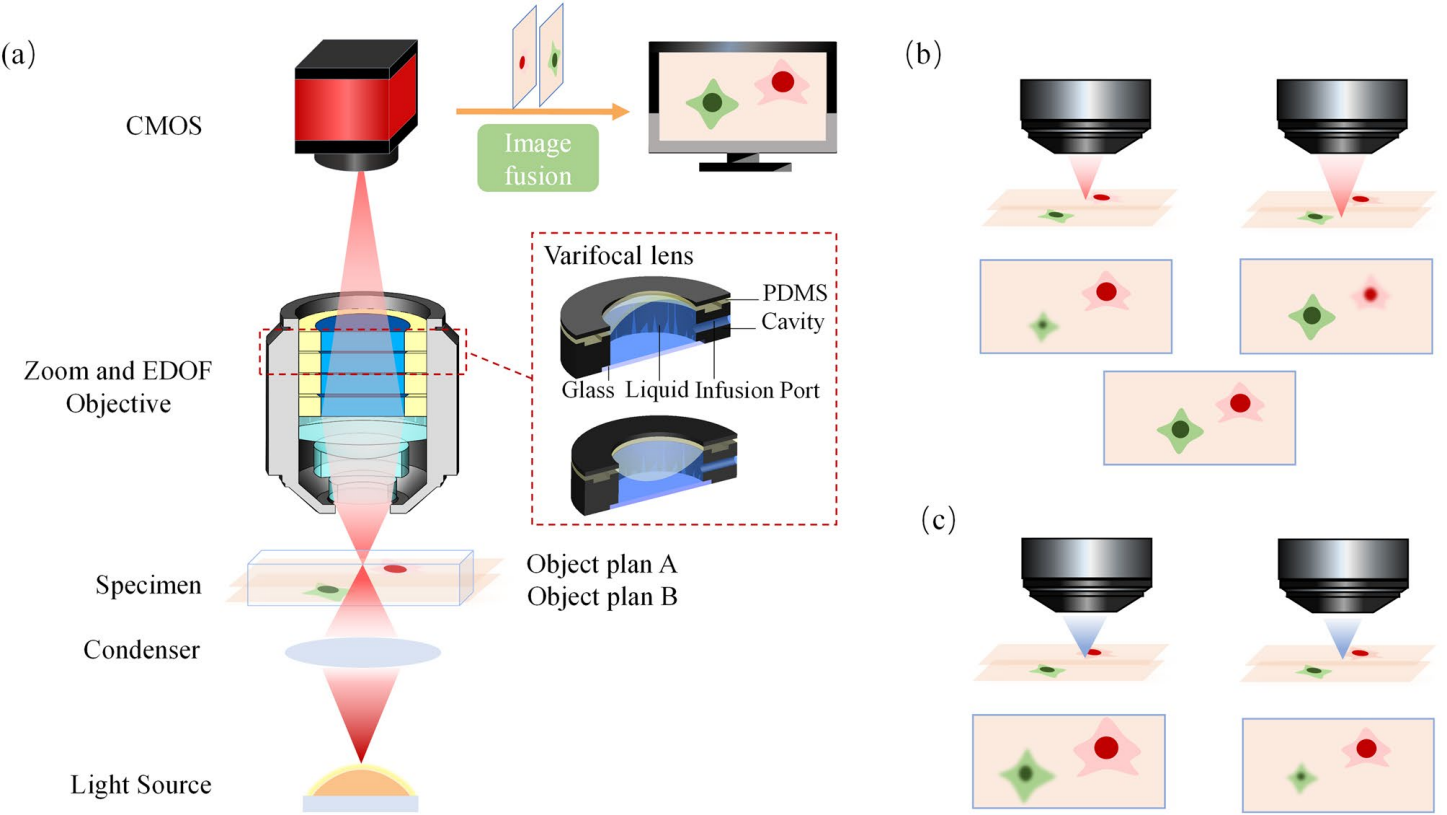
Schematic of the proposed microscope. (**a**) Structure of the proposed microscope and varifocal lens. (**b**) The EDOF function of the proposed microscope. (**c**) The zoom function of the proposed microscope.

Schematic illustration of the proposed microscope is shown in Fig. 1a, which consists of four major components: an EDOF and zoom microscope objective, an image sensor, a lighting system and an image processing system. As the essential element, the varifocal lens is specially designed and fabricated to help the system achieve not only continuous optical zoom with a large zoom ratio, but axial scanning with constant magnification. Combined with the proposed image fusion algorithm, the proposed microscope can realize two functions: EDOF and optical zoom. For the EDOF function, the proposed microscope can realize EDOF with constant magnification and high resolution. By varying the curvature of the varifocal lenses, a series of 2D images with same magnification are obtained, and then processed by the proposed image algorithm rapidly to generate the EDOF image. as shown in Fig. 1b. The optical zoom function is shown in Fig. 1c. The combination of the varifocal lenses is optimized to achieve different magnifications without any mechanical movement. Thus, the proposed microscope can obtain the EDOF images at any magnifications in real time without mechanical movement, which is not available for existing microscopes.

**Figure 2:**
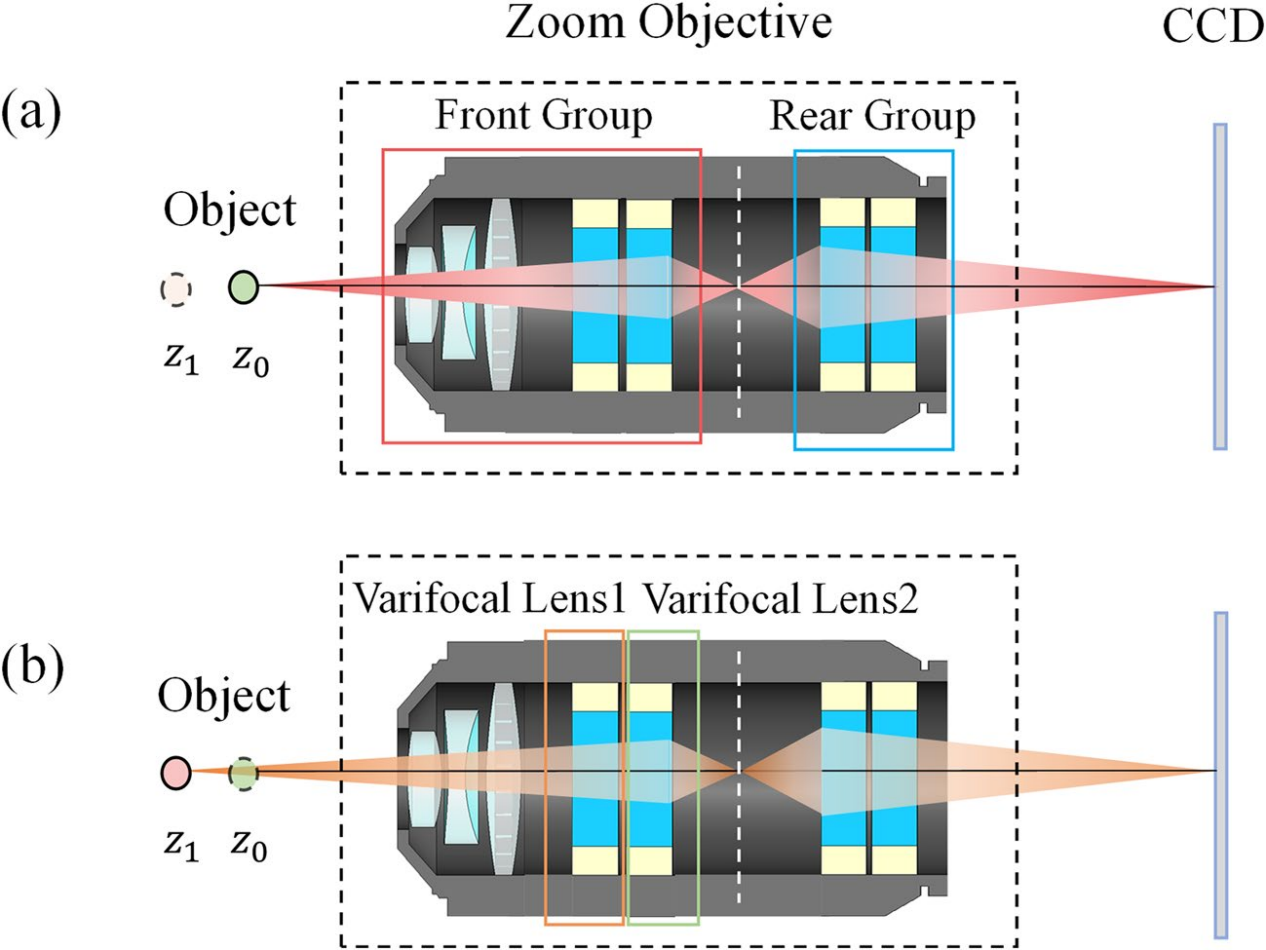
Simplified schematic diagram of the proposed microscope objective. (**a**) Focusing on the plane $z_{0}$. (**b**) Focusing on the plane $z_{1}$.

As the key part of the proposed microscope, the EDOF and zoom microscope objective consists of several glass lenses and four varifocal lenses. Simplified schematic diagram of the microscope objective is shown in Fig. 2a. Four varifocal lenses provide four variables to realize optical axial scanning, zooming, and keeping constant magnification with high resolution. In this way mechanical displacement of the sample during scanning and zooming can be avoided. For conventional optical sectioning techniques, the magnification of the acquired image varies with the scanning depth, and it is difficult to correct the problem of inconstant magnification by using only one focal length as the variable. However, the introduction of varifocal lenses can increase the degree of freedom of the microscope objective, which can be used to correct the problem of inconsistency when extending the DOF and realize optical zoom.

In order to make full use of the zoom capability of the varifocal lens, we adopt a splicing design of the front and rear groups in the optical path. The image plane of the front group coincides with the object plane of the back group, and the image space NA of the front group matches the object space NA of the rear group. In this way, we can use the front group to increase the NA of the objective, while the rear group is used to expand the zoom ratio. In addition, both the front and rear groups have zoom capability, so they can work together to achieve continuous optical zoom by controlling the curvature of the four varifocal lenses. In addition, the change of the magnification $M_{{\text{Front }}}$ of the front group is caused by the change of the object distance, and the change of the magnification $M_{{{\text{Rear}}}}$ of the rear group is caused by the change of the image distance. The optical distance of the front and rear groups remains unchanged, so the equation of zoom magnification can be obtained as follows,

1 $$ \frac{{1 - M_{Front}^{2} }}{{M_{Front}^{2} }}f_{Front}^{\prime } dM_{Front} + \frac{{1 - M_{{{\text{Re}} ar}}^{2} }}{{M_{{{\text{Re}} ar}}^{2} }}f_{{{\text{Re}} ar}}^{\prime } dM_{{{\text{Re}} ar}} = 0, $$

where $M_{{{\text{Front}}}}$, $M_{{{\text{Rear}}}}$ are magnifications of the front group and rear group of the proposed objective, respectively. Besides, $f_{{{\text{Front}}}}^{\prime }$, $f_{{{\text{Rear}}}}^{\prime }$ are the focal length of the objective, the front group and rear group, respectively.

The transverse magnification of the microscope objective ($M_{{{\text{Ob}}}}$) can be given by the below equation.

2 $$ M_{{{\text{Ob}}}} = M_{{{\text{Front}}}} * M_{{{\text{Rear}}}} , $$

Considering the characteristics of the front and rear group in the light path, we use the two varifocal lenses in the front group as the focusing group to extend EDOF of microscope and keep constant magnification. As shown in Fig. 2b, By adjusting the curvature of the two varifocal lenses, microscope objective focus quickly in a certain depth, extending the DOF of microscope from $z_{0}$ to $z_{1}$. Since there is only one surface with variable curvature in the varifocal lens, we can simplify it to a simple plano-convex lens for ease of calculation. To keep magnification of the proposed objective constant, the focal length of two varifocal lenses are required to satisfy Eq. (3).

3 $$ \frac{{f_{P11}^{\prime } }}{{f_{P21}^{\prime } }} = \frac{{z_{1} - f_{G}^{\prime } }}{{z_{0} - f_{G}^{\prime } }} * \frac{{f_{P12}^{\prime } }}{{f_{P22}^{\prime } }}, $$

where $f{^{\prime}}_{P11}$, $f{^{\prime}}_{P21}$, $f{^{\prime}}_{P12}$ and $f{^{\prime}}_{P22}$ are the corresponding focal lengths of varifocal lens 1 and lens 2 when the focusing distances of objective are z0 and z1, respectively. $f{^{\prime}}_{G}$ is the focal length of glass lens group.

According to the theoretical analysis above, the proposed objective can realize continuous optical zoom and extending the DOF with constant magnification based on specially designed parameters.

## Structure and properties of the varifocal lens

**Figure 3:**
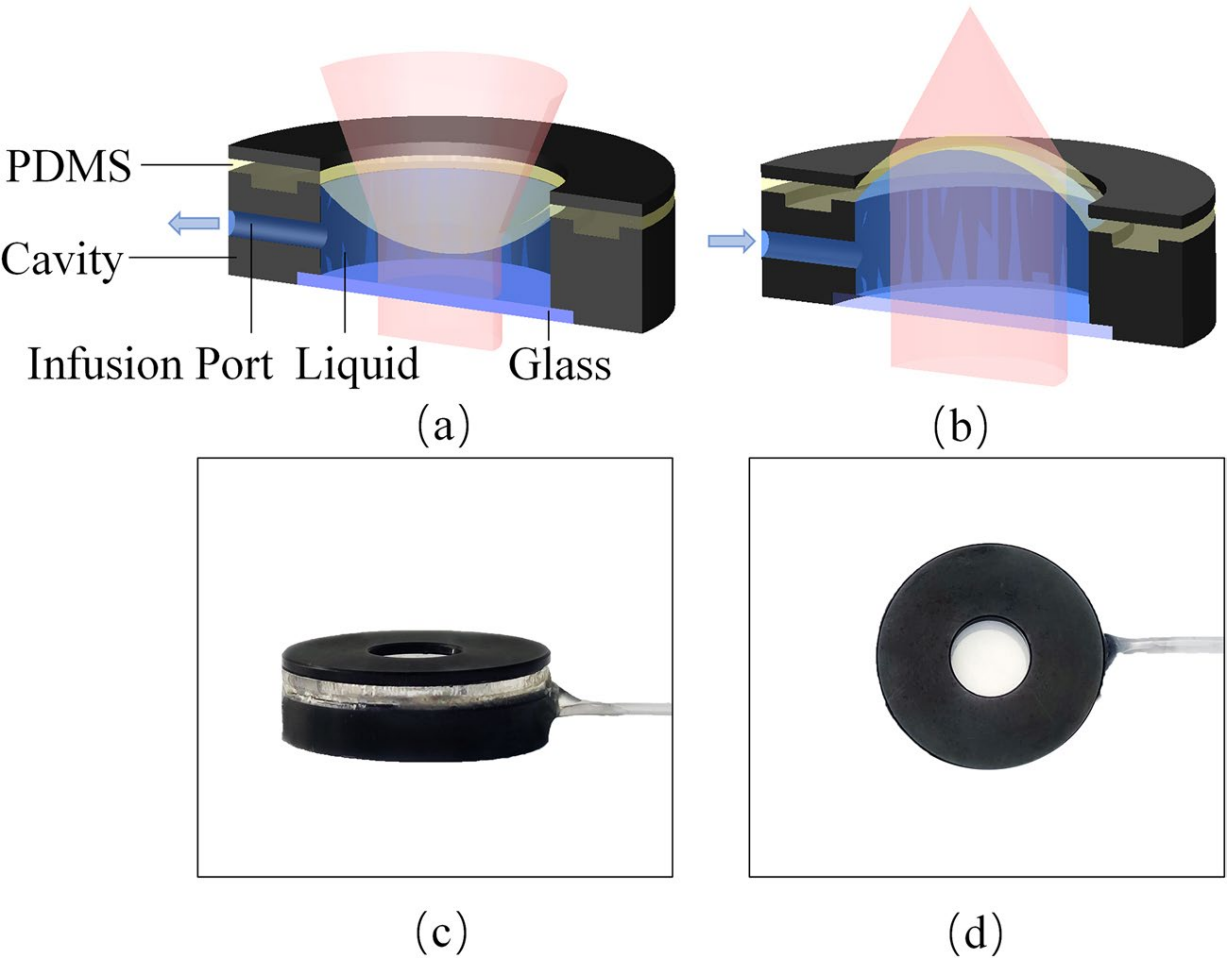
The schematic diagram and physical image of the proposed PDMS lens. (**a**) Divergence mode of the proposed PDMS lens. (**b**) Focus mode of the proposed PDMS lens. (**c**) Side view of the proposed PDMS lens. (**d**) Top view of the proposed PDMS lens.

To achieve high resolution and large tuning range, we designed the varifocal lens as shown in Fig. 3. The proposed varifocal lens is composed of the PDMS film, cavity, optical liquid, a glass and an external liquid pump driver, so it can be called PDMS lens. The curvature of the PDMS lens can be changed by controlling the volume of liquid injected, the focal length of PDMS lens can be changed accordingly. As shown in Fig. 3a, when pumping out liquid, PDMS film dents inward. In this state, the PDMS lens is a negative lens and diverts light. On the contrary, PDMS film protrudes outwards when injecting liquid, and the PDMS lens converges light, as shown in Fig. 3b. The side view and top view of the fabricated PDMS lens are shown in Fig. 3c and d, respectively. The size of PDMS lens is 4 mm (height) × 18 mm (diameter). The effective aperture is 10 mm. Filled liquid is NaCl solution, which has a density of 1.17 g/cm^3^ and refractive index of 1.372.

Assuming that the PDMS lens is placed horizontally, gravity will affect the shape of the film, which can be expressed by Eq. (4)^29^.

4 $$ \delta {\text{gravity = }}A_{p} \left( {1 - v^{2} } \right)\frac{{\rho d^{5} }}{{Et^{3} }} $$

where v, ρ, d, and t are respectively the Poisson’s ratio, density of liquid, diameter of liquid lens and the thickness of membrane. $A_{p}$ is a coefficient depending on the distribution of the hydraulic. E represents Young’s module.

Theoretically, we can reduce this deformation by controlling the film configuration ratio, appropriately reducing film thickness, using middle area of the film and so on. In fact, the varifocal lenses in the proposed objective are placed horizontally in actual use, so the effect of gravity can be ignored, and the surface of proposed varifocal lens can be considered as spherical.

Since the deformation of the PDMS lens can be regarded as a spherical surface, we can deduce the relationship between the increment of the injected liquid ($\Delta V$), the effective diameter of liquid lens (D) and the radius of curvature of the PDMS lens (R), which is shown as Eq. (5).

5 $$ \Delta V = \frac{1}{3}\pi \left( {2R^{2} - D^{2} - 2R\sqrt {\left( {R^{2} - D^{2} } \right)} } \right) \times \left( {2R + \sqrt {R^{2} - D^{2} } } \right) $$

The proposed PDMS lens can be regarded as a plano-convex lens or plano-concave lens, so the relationship between focal length and curvature of PDMS lens (R) can be simplified to the Eq. (6).

6 $$ f = \frac{R}{{n_{2} - n_{1} }} $$

where $n_{1}$ and $n_{2}$ represent the refractive indexes of index of the filled liquid and medium, respectively.

**Figure 4:**
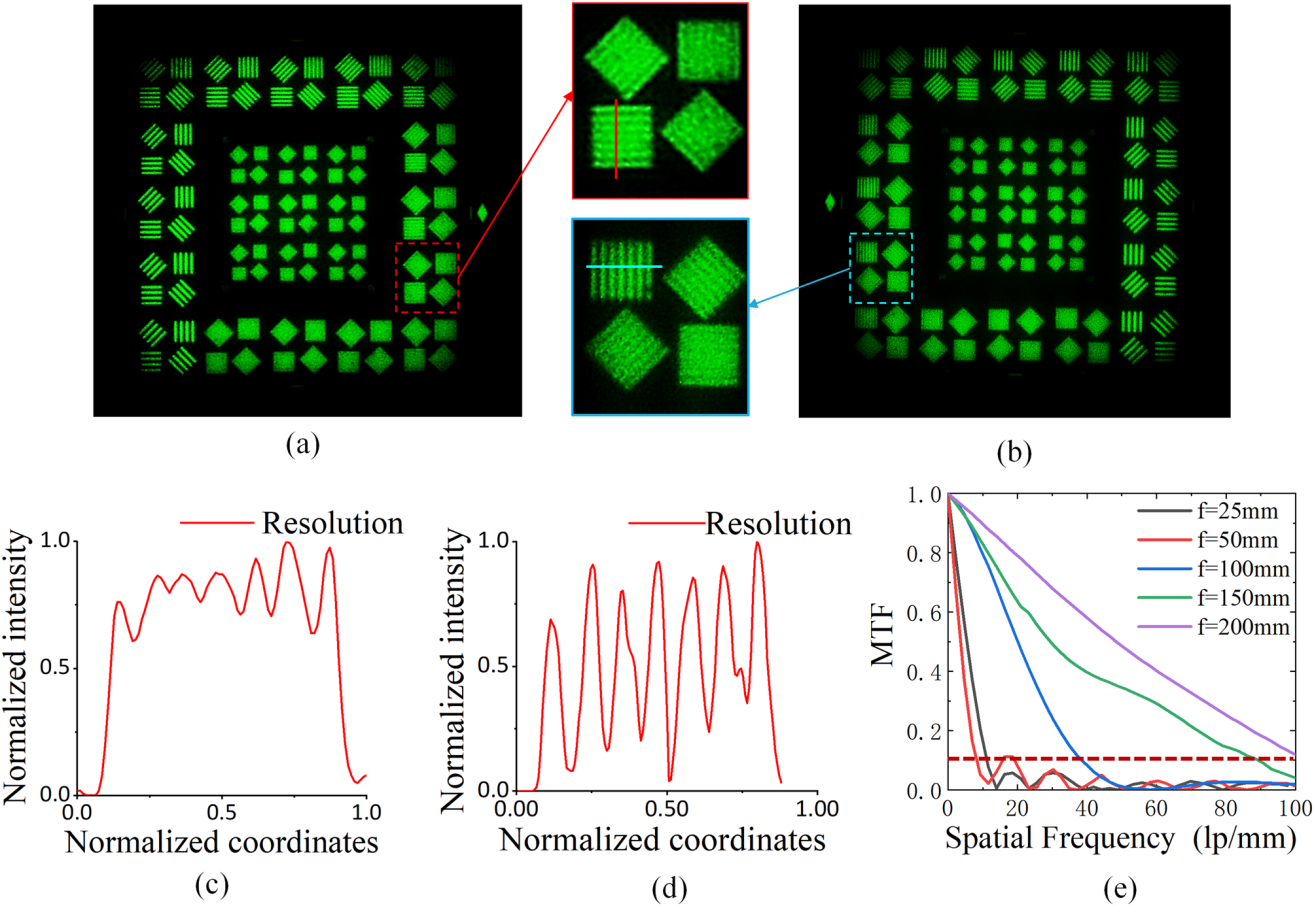
Captured image of JB/T9328-1999 resolution target. (**a**) Focal length is 50 mm. (**b**) Focal length is 150 mm. (**c**) Focal length is 50 mm and resolution of 6 lp/mm. (**d**) Focal length is 150 mm and resolution of 6 lp/mm. (**e**) MTF of PDMS lens at different focal length.

The imaging quality of the proposed PDMS lens is tested as shown in Fig. 4. The captured image by the PDMS lens at f = 50 mm and f = 150 mm are shown in Fig. 4a and b, respectively. The resolution target is JB/T9328-1999. Besides, the resolution at line pair pattern of 6 lp/mm can be calculated as shown in Fig. 4c and d respectively. From the test results we can conclude that the resolution is 6 lp/mm at least. The MTF of the PDMS lens at different focal length is illustrated in Fig. 4e, which shows that the resolution of the proposed PDMS lens is high at different focal lengths.

**Figure 5:**
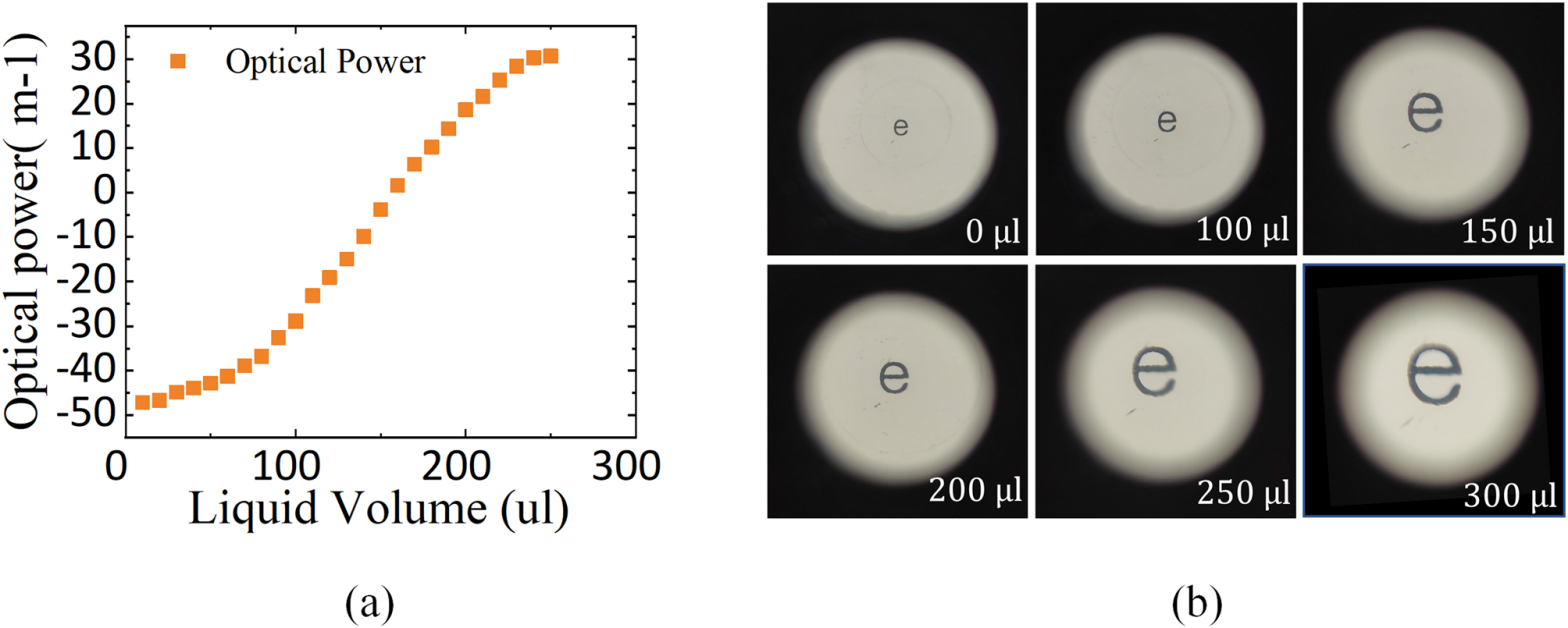
Focusing characteristics of the proposed PDMS lens. (**a**) Correspondence between injected liquid volume and optical power of the proposed PDMS lens. (**b**) Capture imaging of “e” specimen under different liquid volume.

Focusing characteristics of the proposed PDMS lens are shown in Fig. 5. Figure 5a plots the correspondence between injected liquid volume and optical power of the proposed PDMS lens. It can be seen as the volume of injected liquid increases, optical power of the PDMS lens increases. The range of optical power is quite large, up to − 50 m^−1^–32 m^−1^. In addition, when the optical power is small, the correlation between the optical power of the PDMS lens and the injected liquid volume is close to a linear relationship. The capture images of “e” specimen under different liquid volume are shown in Fig. 5b, on which the letters are clearly identifiable. It verifies that the PDMS lens has good imaging quality. However, there are some chromatic aberrations and distortions at the edge of the field of view as the optical power of the PDMS lens increases. In this case, we can try to use the intermediate focal length of the PDMS lens.

## Properties of the EDOF and zoom objective

**Figure 6:**
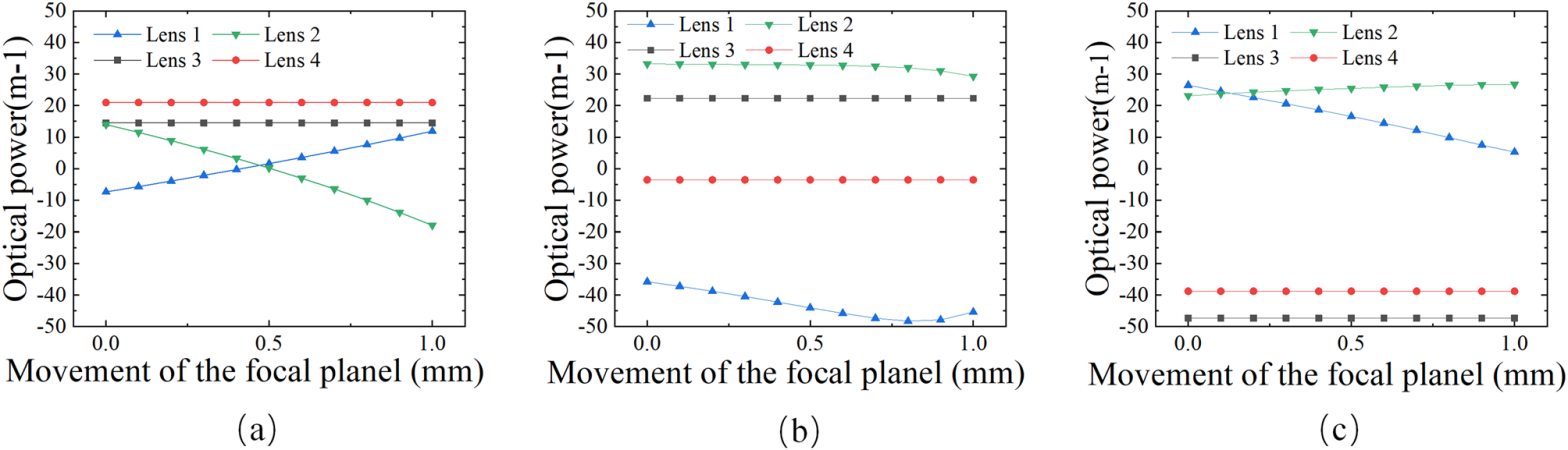
Corresponding relationship between optical power of the proposed varifocal lens and the movement (z) of the focal plane at different magnifications (M). (**a**) 10×. (**b**) 20×. (**c**) 40×.

In order to achieve high resolution, the EDOF objective is optimized at different magnifications and different focal distances. A 2/3’’ CMOS is selected as the image sensor. The largest diagonal field-of-view (FOV) of the EDOF microscope objective is 0.55 mm (V) in the object space. In addition, polychromatic wavelengths of 486 nm, 587 nm and 656 nm are set in the lens model. By adjusting the curvature of the four varifocal lenses we can get the zoom range of the objective from 10× to 40×. The corresponding relationship between optical power of proposed varifocal lens and the movement (z) of the focal plane at different magnifications (M) are shown in Fig. 6. Figure 6a shows that the optical power of four varifocal lenses when the focal plane of objective moves from *z* + 0 mm to *z* + 1 mm. Additionally, as shown in Fig. 6b and c, when the magnifications are 10×, 20× and 40×, respectively, we can control the lens1 and lens 2 in the focus groups to change the focal distance from *z* + 0 mm to z + 1 mm, and keep the magnification constant.

**Figure 7:**
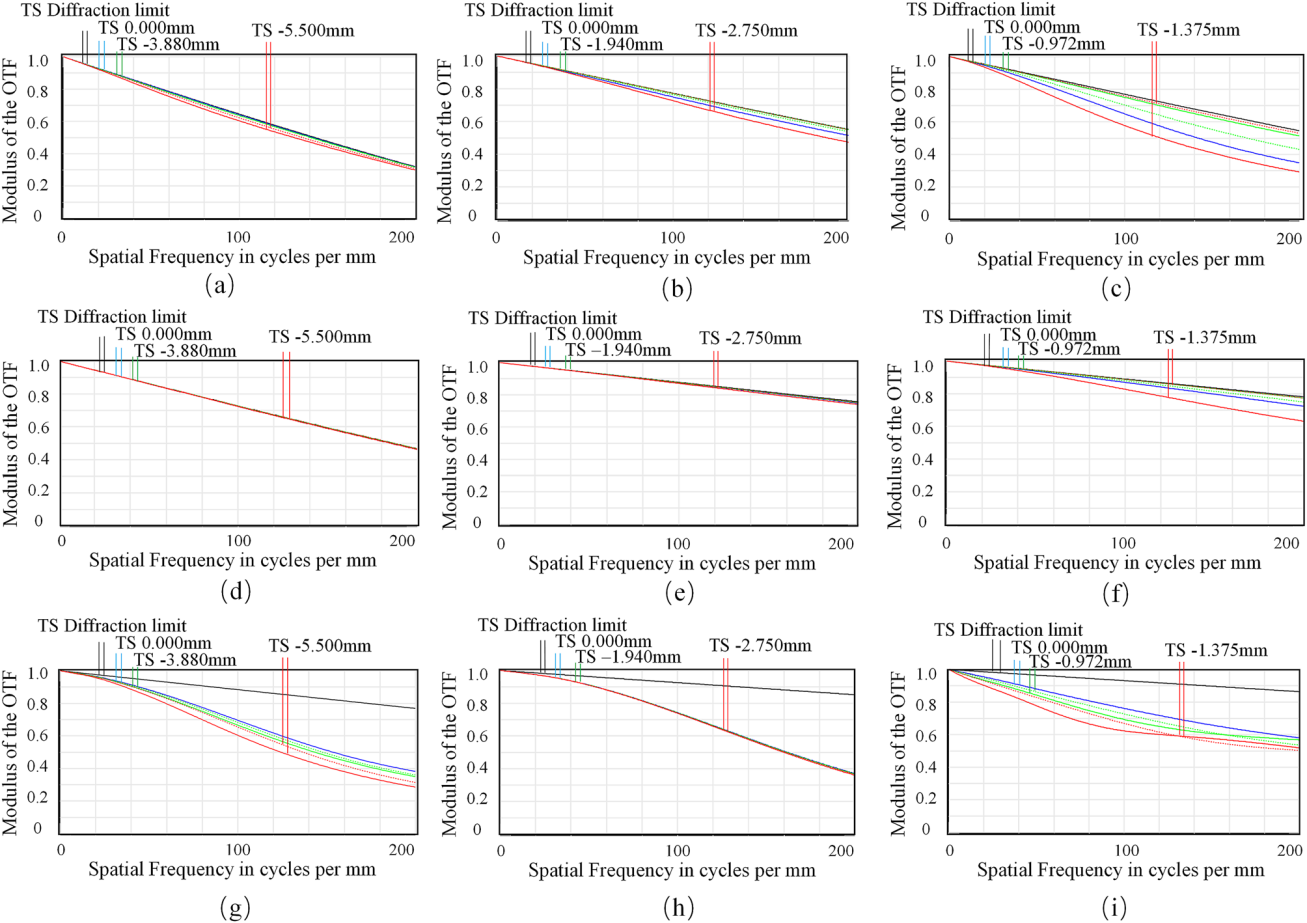
The corresponding diffraction MTF at different magnifications (M) and focal distance (z). (**a**) M = 10×, z = 1 mm. (**b**) M = 10×, z = 3 mm. (**c**) M = 10×, z = 3.5 mm. (**d**) M = 20×, z = 1 mm. (**e**) M = 20×, z = 3 mm. (**f**) M = 20×, z = 3.5 mm. (**g**) M = 40×, z = 1 mm. (**h**) M = 40×, z = 3 mm. (**i**) M = 40×, z = 3.5 mm.

The corresponding diffraction MTF at different magnifications (M) and focal distance (z) are shown in Fig. 7. Figure 7a–c show the MTF when the focal distance (z) of the objective is 1 mm at 10×, 20× and 40×, respectively. When the largest object resolution is 200 lp/mm, the modulus of the OTF at different magnifications are all beyond 0.3. Besides, as shown in Fig. 7d–f, when the focal plane of the objective is moved to (z = 1) mm and (z = 3.5) mm, the resolution at 20× has always been great, although the modulus of the OTF at 10× and 40× have changed greatly, they both are above 0.3. Therefore, we can conclude that the DOF of the proposed microscope objective is extendable to 2.5 mm at 10–40× with high resolution at all working magnifications.

## Experiments and results

**Figure 8:**
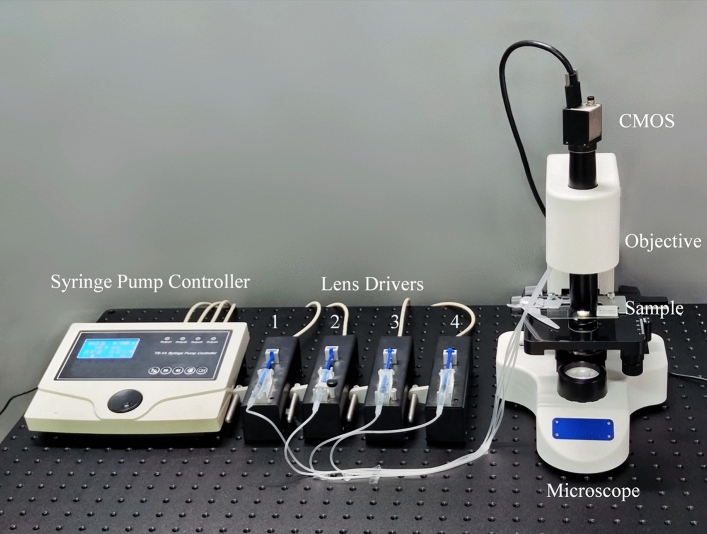
Experimental setup of the EDOF microscope.

**Figure 9:**
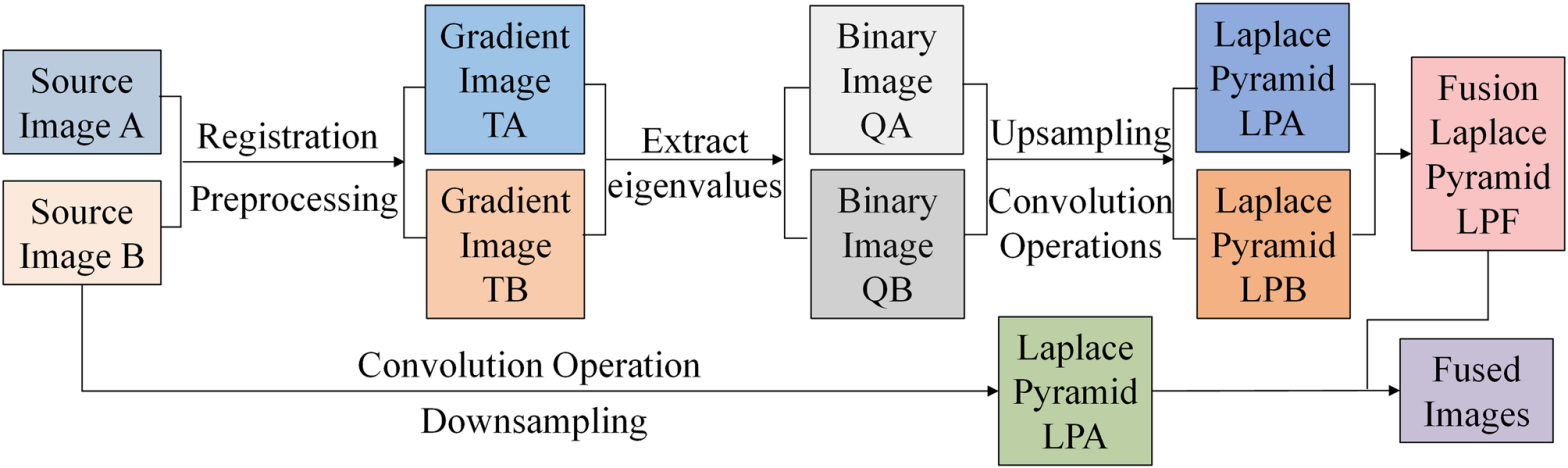
Multifocal image fusion algorithm.

The fabricated EDOF and zoom microscope is shown in Fig. 8. The proposed microscope system consists of the microscope and an external driver system. The proposed EDOF and zoom objective has been integrated into the microscope. The image sensor we use is a 2/3’’ progressive scan CMOS (ON Semiconductor Python 2000) with 1920 × 1200 pixel resolutions. As for the external driver system four independent drivers are controlled by a four-channel syringe pump controller to drive the varifocal lenses respectively. In addition, the algorithm used for synthesizing EDOF image is the improved Laplacian pyramid fusion method, of which the flow chart is shown in Fig. 9. Simply put, two images with different focus are preprocessed to generate gradient images and binary images, which are decomposed into pyramids and fused. The specific fusion rules are: for the top-level image, the pyramid of the fusion image takes the layer with the largest average gradient, and the other layers are fused by the weighted coefficient method, and the coefficient is calculated by the average gradient.

**Figure 10:**
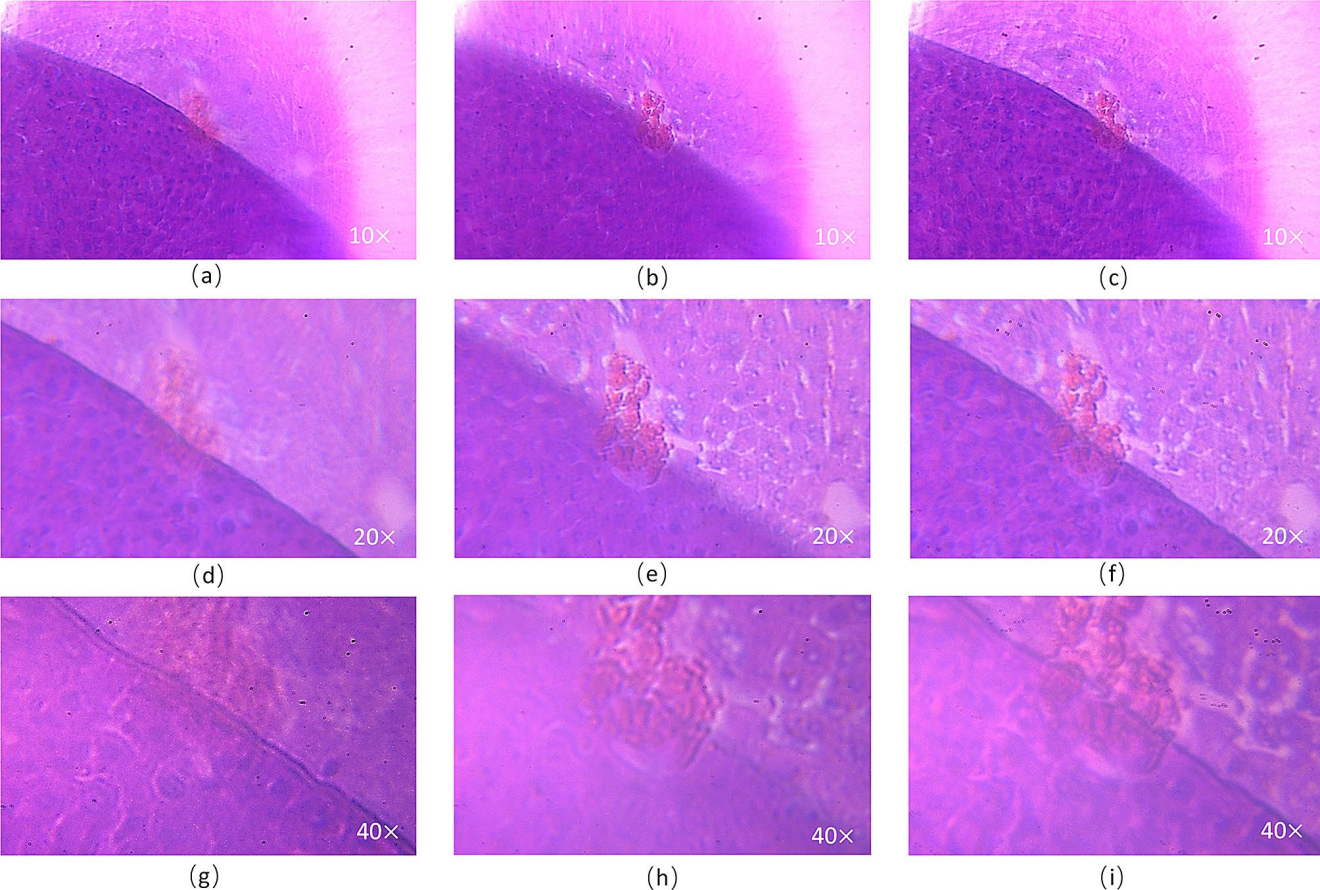
EDOF microscopic images of the mouse lung slices and composite EDOF image at different magnifications. (**a**) Image with 10× objective focused at near distance. (**b**) Image with 10× objective focused at far distance. (**c**) EDOF image at 10×. (**d**) Image with 20× objective focused at near distance. (**e**) Image with 20× objective focused at far distance. (**f**) EDOF image at 20×. (**g**) Image with 40× objective focused at near distance. (**h**) Image with 40× objective focused at far distance. (**i**) EDOF image at 40×.

In the first experiment, we tested the actual imaging quality of the proposed EDOF microscope in a large DOF at different magnifications. The depth of imaged sample was up to approximately 0.32 mm, which was measured by manually translating the sample mounted on the micrometer stage. In the experiment we recorded 4 object plane images with different axial depths for each magnification, and the experimental results are demonstrated in Fig. 10. Firstly, when the initial magnification of the microscope is 10×, the focal distance of the microscope objective is dynamically changed by adjusting the focus group. As shown in Fig. 10a, where the microscope objective focuses at near distance, only the details of the upper slices in the overlapping portion are sharper while the lower slices are strongly blurred due to defocus. In Fig. 10b, only the lower slice is clear after the objective shifts the focus to the corresponding far distance. Figure 10c is a 10× EDOF image synthesized from the collected series of images according to the algorithm shown in Fig. 9. From the results, we can see that the details in both the upper and lower layers are very clear, which indicates that the proposed microscope can achieve the EDOF image in real time. Furthermore, by adjusting the curvature of the four PDMS lenses, the collected near and far images and EDOF images of the sample at 20× and 40× are shown in Fig. 10d–i, respectively. It can be seen the synthesized EODF images are sharp in detail from near to far at all magnifications. However, in conventional microscope images, the DOF of the microscope decreases with increasing the magnification, so it is hard to see the upper and lower layers at the same time. This result verifies zoom and EDOF capability of the proposed microscope, showing that the microscope can extent DOF to 320 µm in the continuous zoom range of 10–40×. Besides, the images are all of high resolution and sharp details.

**Figure 11:**
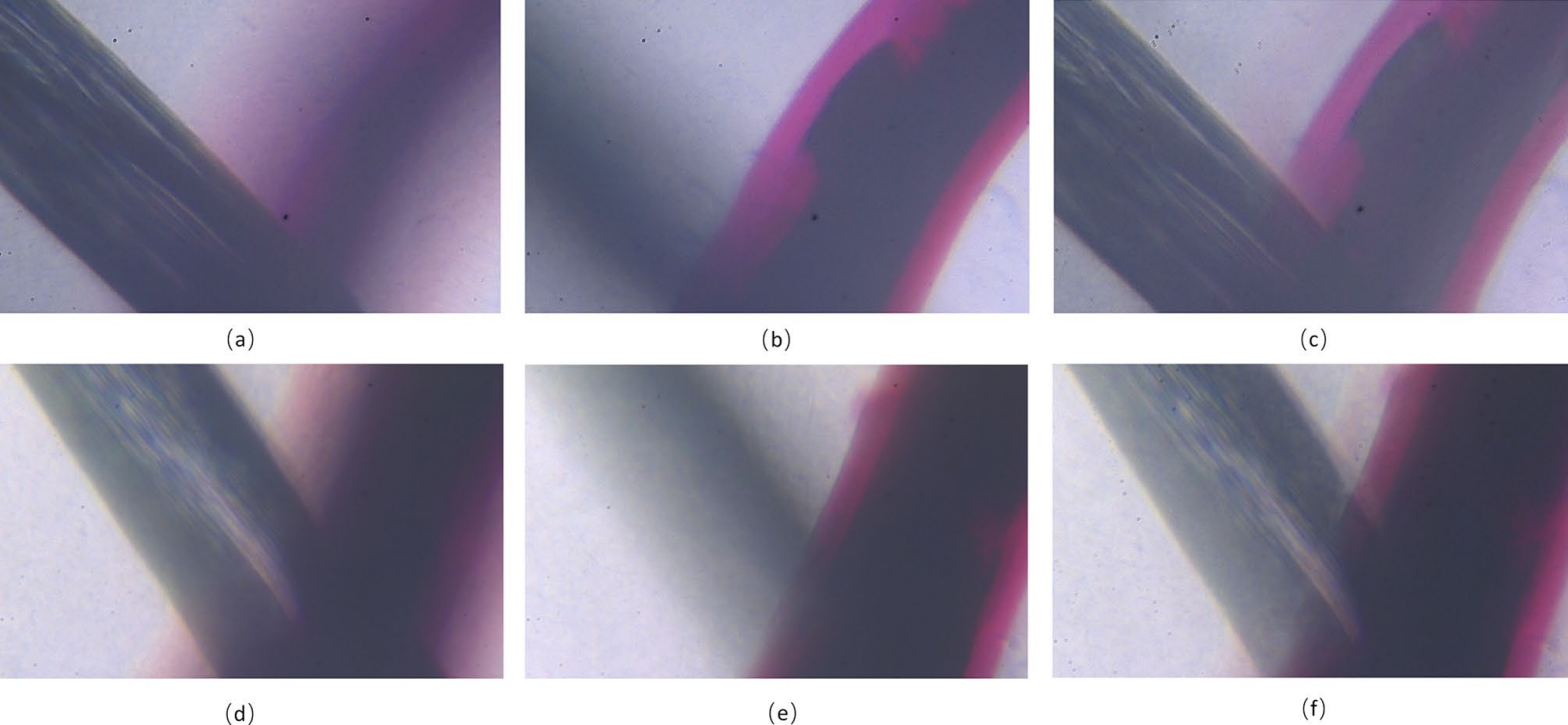
Experiment results of imaging whipworm slices by the proposed microscope and conventional microscope at 20×. (**a**) Image of proposed microscope at near distance. (**b**) Image of proposed microscope at far distance. (**c**) EDOF image of the proposed microscope at 20×. (**d**) Image of conventional microscope at near distance. (**e**) Image of conventional microscope at far distance. (**f**) EDOF image of conventional microscope at 20×.

We further conducted another experiment to verify if the proposed microscope can maintain the same magnification when expanding the DOF. In the experiment, we respectively used the proposed microscope and the conventional microscope^30^ to image the whipworm slices at 20×. The depth of the imaged whipworm slices is up to approximately 1.2 mm, which is measured by manually translating the sample mounted on the micrometer stage. In the experiment we recorded almost 15 process images with different axial depths for each magnification, and the experimental results are demonstrated in Fig. 11. The results imaged by the proposed microscope at different depths of the sample are shown in Fig. 11a and b, respectively, and the synthesized EDOF image shown in Fig. 11c. In contrast, the imaging results of conventional microscope and synthesized EDOF image are shown in Fig. 11d–f, respectively.

Comparing the two synthetic EDOF images shown Fig. 11c and f, we can find that there are discordant colors at the edge of the object in the EDOF image imaged by conventional microscope, which can be considered as the error of image fusion caused by inconstant magnification during axial scanning. While as shown in Fig. 11c, the edge of the observed object is sharp within the required DOF in the EDOF image obtained by the proposed microscope. Therefore, we can conclude that the proposed microscope can realize an extension of the DOF with a constant magnification.

## Conclusion

In this work, we develop an extended the depth of field (EDOF) and zoom microscope, which can realize extension of the DOF with constant magnification and high resolution. Besides, the proposed microscope can achieve optical axial scanning at different NA and magnifications in real time without any mechanical movement. As the core element of microscope, the proposed varifocal lens is employed to realize optical axial scanning, zoom and keeping constant magnification. In addition, experimental results show that the proposed EDOF microscope can achieve a continuous optical zoom in 10–40×, and the DOF can be extended to 1.2 mm with constant magnification. Besides, the objective has a higher degree of freedom to be combined with various microscopy.

## Supplementary Information

Supplementary Information.

## Data Availability

All data generated or analysed during this study are included in this published article (and its supplementary information files).
