# Supplementary material for: Extended the depth of field and zoom microscope with varifocal lens
Source: Sci Rep. 2022 Jun 30;12:11015. doi: 10.1038/s41598-022-15166-x (PMC9247047; doi:10.1038/s41598-022-15166-x)
Supplement: Supplementary file 1 — Supplementary Information. [file 41598_2022_15166_MOESM1_ESM.docx]

**Supplementary material**

**1.Theory of EDOF and zoom microscope objective**

For simplicity, the traditional EDOF microscope system shown in Fig. S1 consists of a conventional microscope objective with infinite conjugate distance, a tube lens, an CCD, and a thick object. In order to obtain the EDOF image, the focal plane of the microscope objective is scanned rapidly through the object from z0 to z1. However, the magnification of the acquired image at this time varies with the scanning depth, the change in magnification $\Delta M$ is given by formula (3). Furthermore, it can be seen from the formula that only one focal length as a variable is difficult to correct the problem of inconsistent magnification.

$\text{Δ}_{M}=\frac{f_{\text{Tube }}^{'}\cdot\text{Δ}z}{f_{Ob}^{'}\left(f_{Ob}^{'}+\text{Δ}_{z}\right)}$,

Where ${f_{Tube}}^{'}$and${f_{Ob}}^{'}$are the focal lengths of the tube lens and the objective, respectively, and$\Delta z$ is the axial movement of the focal plane.

Based on the above-mentioned variable curvature principle of PDMS lens, we consider that part of the traditional glass lens in objective can be replaced with PDMS lenses. In this way objective can realize zooming and optical axial scanning, achieving the purpose of expanding the depth of focal. In addition, the introduction of PDMS lenses increases the degree of freedom of the system, which can be used to correct the problem of inconsistency in magnification

As shown in Fig. S2, two PDMS lenses make up focus groups in the above microscope system to maintain consistent magnification when extending DOF. By adjusting the curvature of the two lenses, the focal plane of the objective is moved from z0 to z1. Since the PDMS lens has only one surface with variable curvature, we can simplify it to a simple plano-convex lens for ease of calculation. It can be known by derivation that to keep magnification of the objective constant, only the focal length of two PDMS lenses are required to satisfy the following relationship (2).

$\frac{f_{P11^{'}}^{'}}{f_{P21}^{'}}=\frac{z_{1}-f_{G}^{'}}{z_{0}-f_{G}^{'}}*\frac{f_{P12^{'}}}{f_{P22^{'}}^{'}}$,

Where ${f_{P11}}^{'}$, ${f_{P21}}^{'}$, ${f_{P12}}^{'}$ and ${f_{P22}}^{'}$ are the corresponding focal lengths of PDMS lens 1 and lens 2 when the focusing distances of objective are z0 and z1, respectively. $f_{G}'$ is the focal lengths of Front lens group.


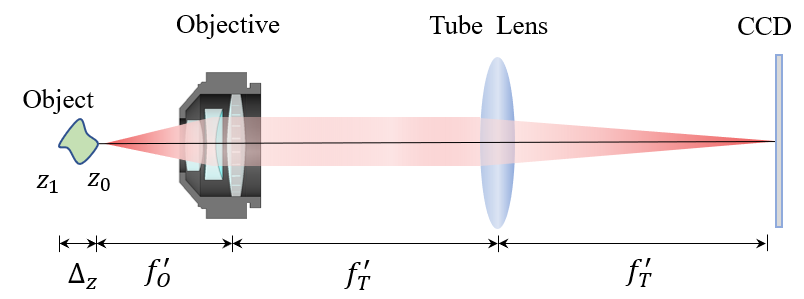


Figure. S1. Schematic diagram of traditional EDOF microscope objective


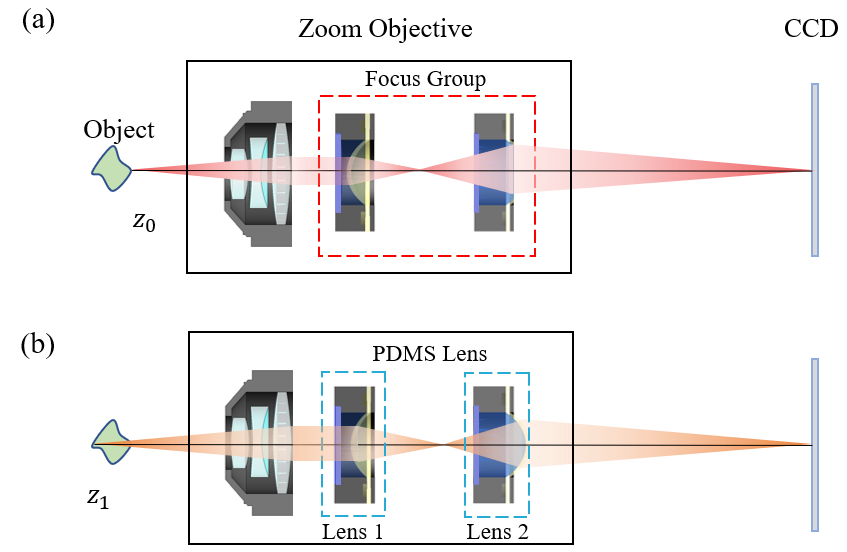


Figure. S 2. Schematic diagram of EDOF microscope objective based on PDMS lens (a) The object plane $z= z_{0}$ (b) The object plane $z= z_{1}$

Figure. S 3. Shows the zoom diagram of EDOF microscope objective based on PDMS lens. In order to make full use of the zoom capability of PDMS, we adopt a splicing design of the front and rear groups in the optical path. Both the front and rear groups have zoom capability, and they can work together to achieve continuous optical zoom by controlling the curvature of the four PDMS lenses. The derivation process of the formula for the zooming of the microscope objective is as follows.

When the magnification of the microscope objective changes, for the front group, the object point does not move. Due to the change of the focal length of lens 1 and lens 2, the principal plane of the front group will move slightly ($dp)$, which will cause the movement of the entire lens group ($\Delta1$):

$\Delta1=m_{\text{Rear}}^{2}\left(1-m_{\text{Front}}^{2}\right)dp$,

where $M_{\mathrm{Front}}$, $M_{\mathrm{Rear}}$ are magnifications of the objective, the front group and rear group, respectively.

For the rear group, the main plane of the rear group will move slightly $dq$. At this time, the amount of movement caused by the entire lens group$\left( \Delta1 \right)$is:

$\Delta2=\left(1-m_{Rear }^{2}\right)dq$,

In order to achieve the stability of the image plane, the algebraic sum of the two image planes should be 0, which can be expressed as the following equation:

$m_{\mathrm{Re}ar}^{2}\left(1-m_{Front}^{2}\right)dp+\left(1-m_{\mathrm{Re}ar}^{2}\right)d\text{q}=0$,

In addition, since the change of the magnification$M_{\mathrm{Front}}$of the front group is caused by the change of the object distance, and the change of the magnification$M_{\mathrm{Rear}}$ of the rear group is caused by the change of the image distance. The optical distance of the front and rear groups remains unchanged, so the equation of zoom magnification can be obtained:

$\frac{1-M_{Front}^{2}}{M_{Front}^{2}}f_{Front}^{'}dM_{Front}+\frac{1-M_{\mathrm{Re}\text{ar}}^{2}}{M_{R\mathrm{e}ar}^{2}}f_{\mathrm{Re}ar}^{'}dM_{\mathrm{Re}ar}=0$,

where ${f'}_{\mathrm{Front}}$, ${f'}_{\mathrm{Rear}}$ are the focal length of the objective, the front group and rear group, respectively.

The transverse magnification of the microscope objective ($M_{\mathrm{Ob}})$can be given by the below formula.

$M_{\text{Ob}}=M_{\text{Front}}*M_{\text{Rear}}$,


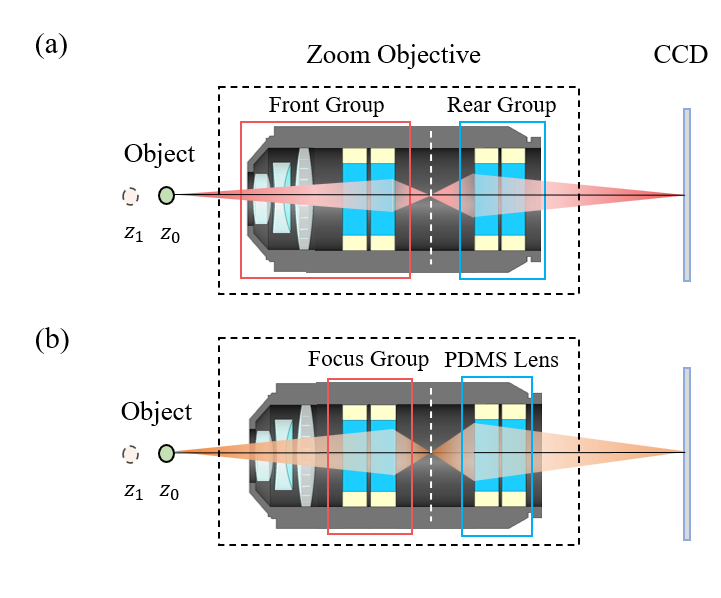


Figure. S 3. Zoom diagram of EDOF microscope objective based on PDMS lens (a) Low magnification. (b) High magnification.

**2. Structure of proposed microscope objective**

The optical path diagram of the proposed microscope objective is shown in the fig. S4. In addition, the detailed parameters of the glass lenses used in the proposed objective are shown in Tab. R1. These will be included in the supplementary materials of the article for reference.


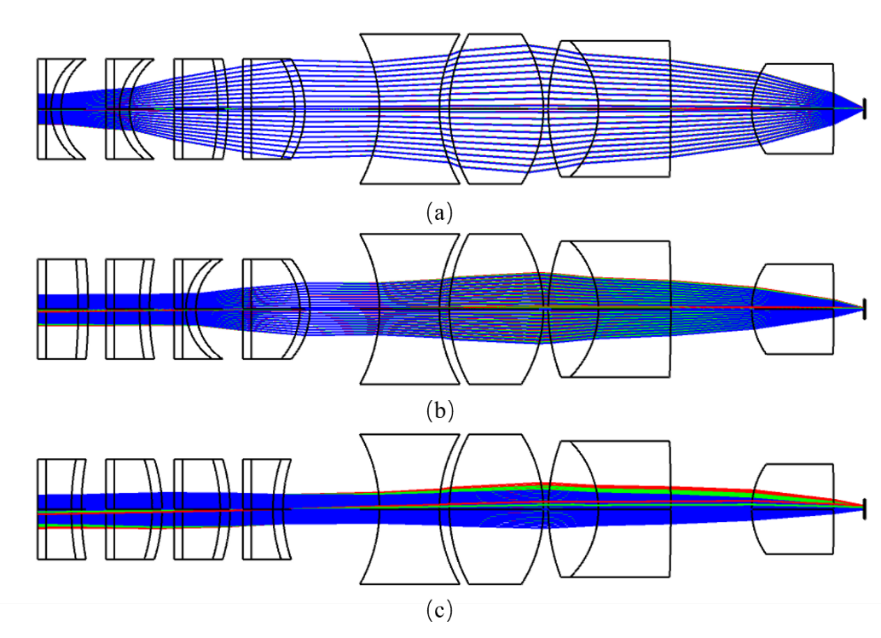


Figure. S4. The optical path diagram of the microscope objective. (a) lay out of the proposed objective at $40\times$. (b) lay out of the proposed objective at $20\times$. (c) lay out of the proposed objective at $10\times$.

Table S1. The detailed parameters of the glass lenses used in the proposed objective.

| \| Surface \| Radius \| Thickness (mm) \| Material \| Clear-Semi-Diameter \| \| --- \| --- \| --- \| --- \| --- \| \| 1 \| 184.93 \| 8.2 \| H-ZK9B \| 4.5 \| \| 2 \| -7.745 \| 8.1 \|  \| 4.5 \| \| 3 \| 199.99 \| 7.25 \| H-ZF10 \| 6.8 \| \| 4 \| 9.594 \| 5 \| H-QK1 \| 6.8 \| \| 5 \| -19.055 \| 0.5 \|  \| 6.8 \| \| 6 \| 13.900 \| 9.4 \| H-K9L \| 7.5 \| \| 7 \| -15.370 \| 1 \|  \| 7.5 \| \| 8 \| -14.723 \| 6 \| H-K9L \| 7.5 \| \| 9 \| 15.370 \| 8 \|  \| 7.5 \| |
| --- | --- | --- | --- | --- | --- | --- | --- | --- | --- | --- | --- | --- | --- | --- | --- | --- | --- | --- | --- | --- | --- | --- | --- | --- | --- | --- | --- | --- | --- | --- | --- | --- | --- | --- | --- | --- | --- | --- | --- | --- | --- | --- | --- | --- | --- | --- | --- | --- | --- | --- |
